# Salt as an Antiseptic

**Published:** 1890-12-13

**Authors:** 


### SALT AS AN ANTISEPTIC.

Professor Fritsch, of Breslau, uses sterilised solution of common salt in abdominal surgery, but the researches of Dr. Freytal undertaken with the view of determining how far the "pickling" of pork was calculated to destroy the germs of disease, have shown that it has no claims whatever to the character of a bactericide except in the case of Koch's cholera bacillus and the growing forms of that of conturax. Doubtless if Fritsch had used pure sterilised water his success would have been as great.

